# Complete mitochondrial genome of *Kusala populi* (Song, Li & Xiong, 2011) (Hemiptera, Cicadellidae, Typhlocybinae) from Karst area, Southwest China

**DOI:** 10.1080/23802359.2023.2209385

**Published:** 2023-05-12

**Authors:** Tianyi Pu, Ni Zhang, Yu Zhang, Yuehua Song

**Affiliations:** a School of Karst Science, Guizhou Normal University/State Engineering Technology Institute for Karst Desertification Control, Guiyang, China; b Guizhou Provincial Key Laboratory for Rare Animal and Economic Insect of the Mountainous Region, Guiyang University, Guiyang, China; c Liuguan Street Middle School, Panzhou, China

**Keywords:** *K. populi*, Mitochondrial genome, phylogenetic analysis, Karst

## Abstract

In this study, we sequenced and reported the complete mitochondrial genomes of *Kusala populi* for the first time. The complete mitochondrial genome was registered in GenBank with accession number NC_064377 as the first complete mitogenome of the genus *Kusala.* The circular mitochondrial genome length is 15,402 bp, with nucleotide composition A (41.8%), C (11.4%), G (9.2%), T (37.6%), A + T (79.4%), and C + G (20.6%), comprising 13 protein-coding genes, 2 rRNA genes, 22 tRNA genes and a D-loop region. All protein-coding genes were encoded by the H-strand, except for 4 genes (*nad5*, *nad4*, *nad4L*, *nad1*). 8 tRNA genes (*tRNA-Gln*, *tRNA-Cys*, *tRNA-Tyr*, *tRNA-Phe*, *tRNA-His*, *tRNA-Pro*, *tRNA-Leu*, *tRNA-Val)* and 2 rRNA genes (*16S*, *12S*) were encoded in the L-strand. Phylogenetic analysis indicated that the newly sequenced species had a close relationship with *Mitjaevia*, another widespread Old-World genus of Erythroneurini.

## 1. Introduction

Leafhoppers, phytophagous insects, are important pests of agriculture, forestry, fruit trees, and other economically important plants (Morris 1971; Guo 2011; Roddee et al. 2018). Cicadellidae is the largest family in Hemiptera, and is distributed all over the worldwide (Zhang et al. 2021). Typhlocybinae is one of the important forestry pests of Cicadellidae. There are eleven species of Oriental genus *Kusala* widely distributed in the world, among them, four are in China, including *Kusala colibria*, *Kusala datianensis*, *Kusala maculata* and *Kusala populi* (Song and Li 2014). *K. populi* (Song et al. 2011) were found in karst areas in Guizhou and Chongqing. However, genetic information on this species has not yet been studied. Thus, we sequenced the mitogenome of *K. populi* to determine the phylogenetic position of the species and the genus *Kusala*. As a first report on the mitogenome of genus *Kusala*, this result will be useful in the future for species identification, phylogenetic studies and biogeographic studies within Typhlocybinae.

## 2. Materials and methods

### 2.1. Sample collection

**Figure 1.**
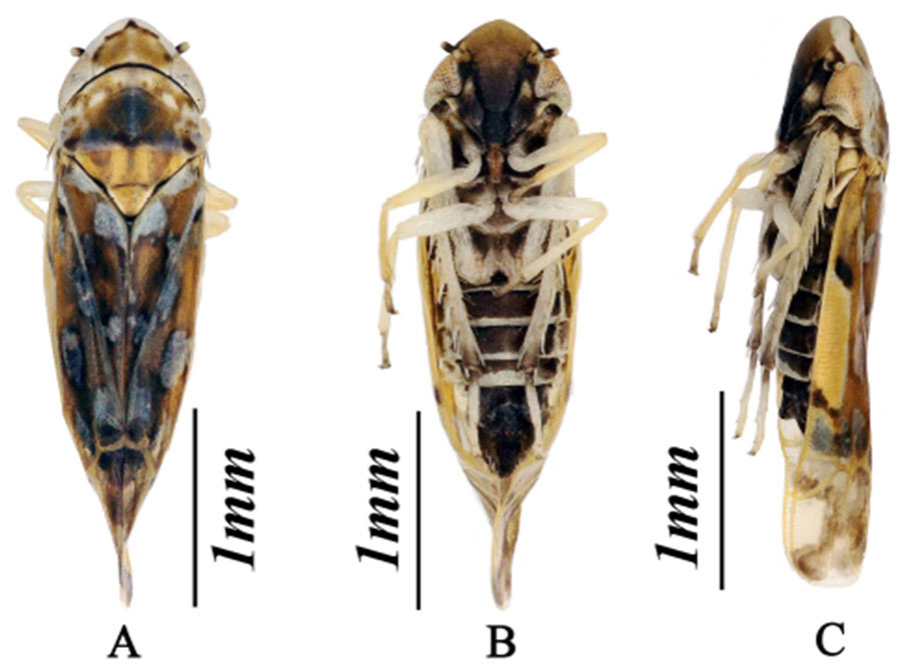
*K. populi* in habitus (A) dorsal view; (B) ventral view; (C) lateral view (The species reference pictures in this paper were taken by the author of this paper on 10 September 2022 at the Biological Laboratory of the School of Karst Science, Guizhou Normal University).

In mid-July 2019, *K. populi* adult specimens (twenty female individuals, ten male individuals, Figure 1) were collected using the sweeping net method from roadside weeds in Huaguan Road, Huaxi District, Guiyang City (26°24′44.90″N, 106°41′33.70″°E, elevation 1171 m). The specimens were morphologically identified, stored in absolute ethanol, and placed in a freezer at −20 °C. The sample and the total DNA were deposited at School of Karst Science, Guizhou Normal University (Voucher number: GZNU-ELS-20190715, http://gznu.edu.cn, contact person: Weiwen Tan; email: 14276371092@qq.com).

### 2.2. Methods

The leafhoppers total DNA was extracted from the entire body of multiple males of the same species (seven individual males of *K. populi*) without the abdomen and wings. Genomic DNA was extracted by using a tissue rapid Extraction Kit (VWI) according to the manufacturer’s instructions. Whole mitochondrial genome sequences were determined at Berry Genomics (Beijing, China) using an Illumina Novaseq 6000 platform (Illumina, Alameda, CA, USA) by 150 bp paired-end reads and 5.87 G of raw data were obtained. Mitochondrial genome assembly was performed using GetOrganelle 1.7.5 (parameters: -R 10 -k 21,45,65,85,105 -F animal_mt) (Jin et al. 2020), and the assembly results were blastn (version: BLAST 2.2.30+; parameters: – evalue 1e-5) with the proximal reference mitogenome (accession number NC_047465), and the candidate sequence assembly results were determined based on the comparison. Gene annotation of the assembled mitogenome was performed using MITOS 2 (Alexander et al. 2019). The annotated mitogenome was deposited in GenBank with the accession number NC_064377. In order to understand the phylogenetic status of *K. populi*, we downloaded 20 closely related mitogenomes from the NCBI database, the *Homalodisca coagulate* and *Cicadella viridis* mitogenomes were selected as the outgroup (GenBank no. AY875213 and no. MK335936). Maximum likelihood (ML) method in IQ-TREE 2.0 (Minh et al. 2020; 2000 ultrafast bootstrap) based on the best-fit model GTR + I + R and Bayesian inference (BI) method in MrBayes 3.2 (Ronquist et al. 2012) were adopted to construct phylogenetic tree using the complete mitochondrial genes.

## 3. Results

**Figure 2.**
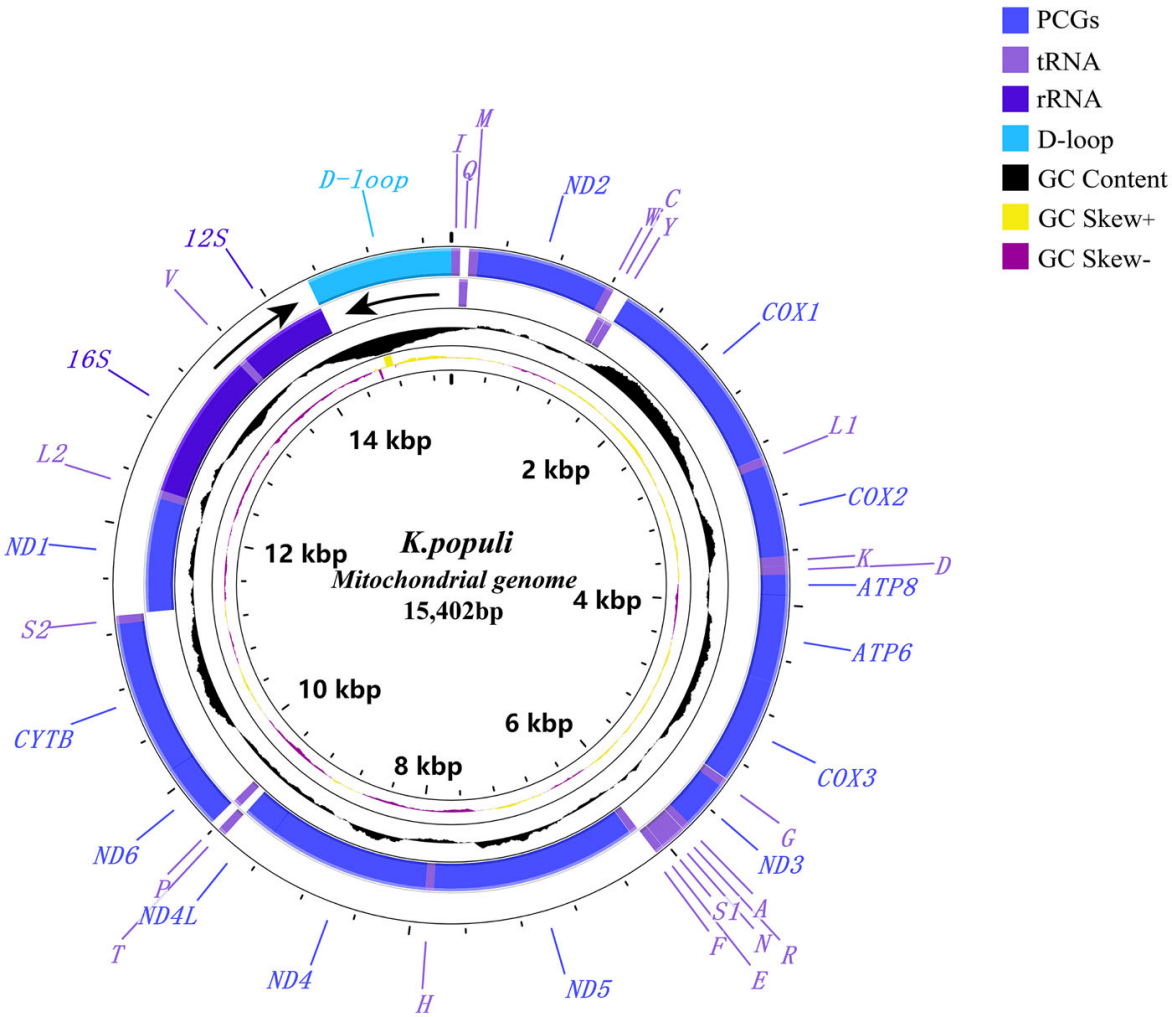
Circular map of the mitochondrial genome of *K. populi.*

**Figure 3.**
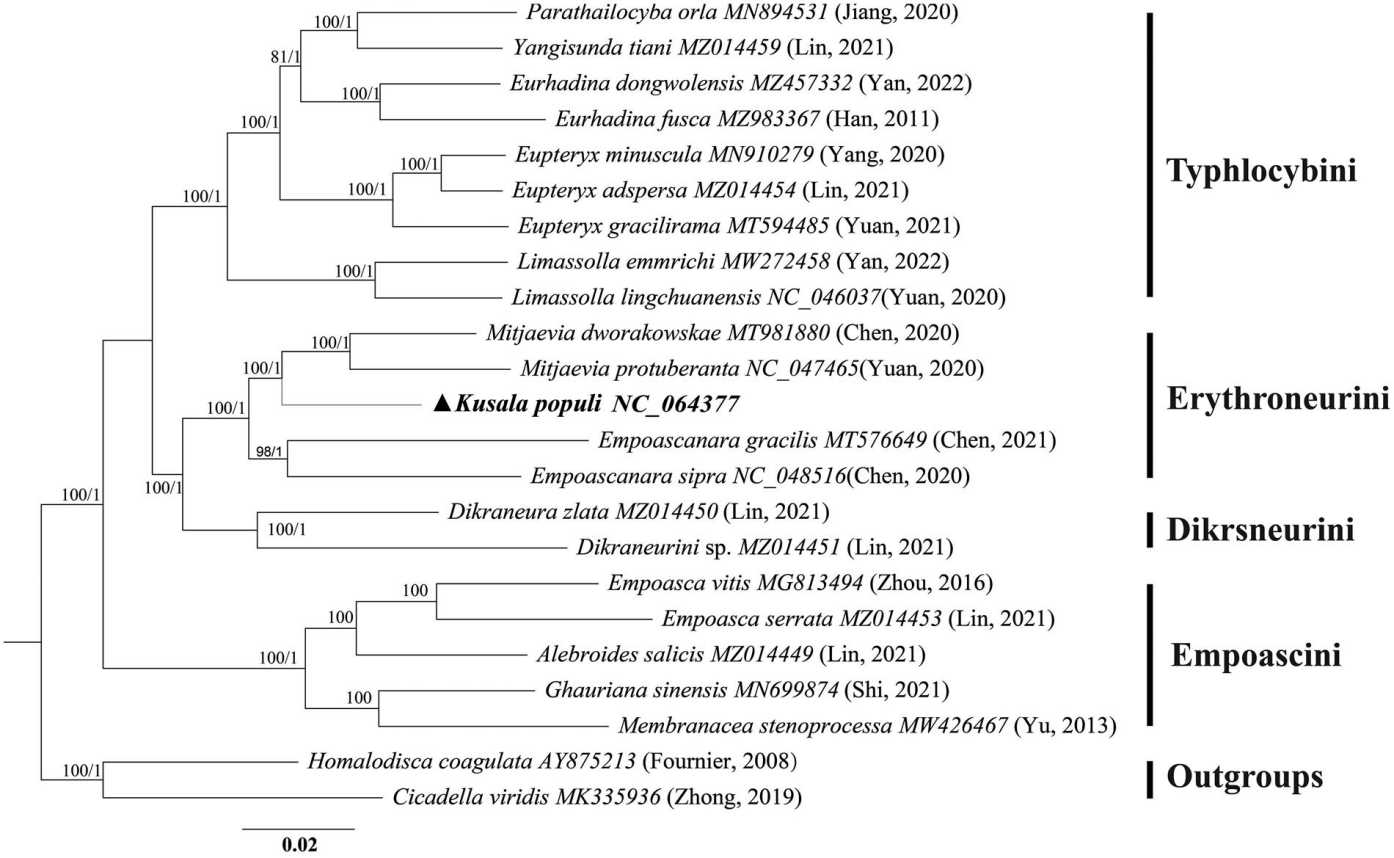
Phylogenetic tree based on 23 species reconstructed using mitochondrial 13PCGs. The first and second numbers to the left of each node denote the ultrafast bootstrap value (UBP) for ML analysis and the Bayesian posterior probability (BPP) for Bayesian inference.

The mitochondrial genome of *K. populi* was 15,402 bp in length, containing 13 protein-coding genes (PCGs), 22 tRNAs, 2 rRNA genes, and a D-loop region (Figure 2). The nucleotide composition of the genome is A (41.8%), C (11.4%), G (9.2%), T (37.6%), A + T (79.4%), and C + G (20.6%). There were 14 genes encoded in the L-strand, and the remaining genes were encoded in the H-strand. The largest gene was *nad5* and the smallest was *atp8* in PCGs. The AT content in PCGs was slightly lower than that in the whole genome. All PCGs start from the ATN (ATA/ATT/ATG) codon except *atp8* where it was TTG and end with the complete stop codon TAA or TAG, TAA was the most frequently used stop codon. The *cox2*, *nad1*, and *nad4* have an incomplete stop codon T–, and the tRNA structure of *K. populi* is similar to that of other Hemiptera (Yuan et al. 2021). The 21 tRNAs can be folded into a typical cloverleaf secondary structure, except *trnS1*, which lacks the dihydrouridine (DHU) stem and forms a simple loop. Phylogenetic analysis indicated that *Kusala* belongs to the tribe Erythroneurini, a sister clade to *Mitjaevia*, i.e. ((*Kusala + Mitjaevia*) *+ Empoascanara*) (Figure 3) (Figure 3; UBP/BPP= 100/1.00).

## 4. Discussion and conclusion

The mitochondrial genome of *K. populi* was 15,402 bp in length. It contained 13 protein-coding genes (PCGs), 22 tRNAs, 2 rRNA genes, and a D-loop region (Figure 2). The analysis showed that the genomic structure of *K. populi* is similar to previously studied (Chen et al. 2021). Reconstructed phylogenetic trees based on 21 subfamily Typhlocybinae species and two outgroups yielded a consistent topology with similar phylogenetic relationships between clades as in previous studies, i.e. (Empoascini + (Typhlocybini + (Erythroneurini + Dikrsneurini))). Our study reveals for the first time the phylogenetic position of *Kusala* within the tribe Erythroneurini and suggests a closer genetic relationship with *Mitjaevia*, which is consistent with the results of Song et al. (2011). Our phylogenetic tree also shows the robustness of the mitochondrial genome in resolving phylogenetic relationships at the tribe and genus level due to the high level of support obtained for these nodes (Figure 3; UBP/BPP = 100/1.00). The resultwill contribute to future phylogenetic studies, population genetics, and biogeography within the subfamily Typhlocybinae.

## Ethical approval

The research of the article does not need Ethical approval. No specific permissions were required for any of the collection localities.

## Data Availability

The data that support the findings of this study are openly available in the GenBank of NCBI athttps://www.ncbi.nlm.nih.gov under the accession NC_064377. The associated BioProject, SRA and Bio-sample numbers are PRJNA842561, SRR19414868, and SAMN28677931, respectively.
